# PERK Pathway Activation Promotes Intracerebral Hemorrhage Induced Secondary Brain Injury by Inducing Neuronal Apoptosis Both *in Vivo* and *in Vitro*

**DOI:** 10.3389/fnins.2018.00111

**Published:** 2018-02-28

**Authors:** Chengjie Meng, Juyi Zhang, Baoqi Dang, Haiying Li, Haitao Shen, Xiang Li, Zhong Wang

**Affiliations:** ^1^Department of Neurosurgery & Brain and Nerve Research Laboratory, The First Affiliated Hospital of Soochow University, Suzhou, China; ^2^Department of Neurosurgery, Yancheng First Peoples' Hospital, Yancheng, China; ^3^Department of Rehabilitation Medicine, Zhangjiagang Hospital of Traditional Chinese Medicine, Suzhou, China

**Keywords:** PERK pathway, intracerebral hemorrhage, secondary brain injury, apoptosis, ER stress

## Abstract

The protein kinase R (PKR)-like endoplasmic reticulum kinase (PERK) signaling pathway was reported to exert an important role in neuronal apoptosis. The present study was designed to investigate the roles of the PERK signaling pathway in the secondary brain injury (SBI) induced by intracerebral hemorrhage (ICH) and its potential mechanisms. Sprague–Dawley rats were used to establish ICH models by injecting autologous blood (100 μl), and cultured primary rat cortical neurons were exposed to oxyhemoglobin (10 μM) to mimic ICH *in vitro*. The PERK antagonist, GSK2606414, and inhibitor of eukaryotic translation initiation factor 2 subunit α (eIF2α) dephosphorylation, salubrinal, were used to study the roles of PERK signaling pathway in ICH-induced SBI. Our results showed that the protein levels of p-eIF2α and ATF4 were upregulated following ICH, peaking at 48 h. Application of GSK2606414 reversed this increase *in vivo* and *in vitro*, thereby preventing ICH-induced neuronal apoptosis. On the contrary, salubrinal inhibited the dephosphorylation of eIF2α, resulting in the elevation of p-eIF2α, which could activate downstream of PERK signaling and induce neuronal apoptosis and necrosis following ICH *in vitro* and *in vivo*. Thus, PERK signaling pathway plays an important role in ICH-induced apoptosis and blocking its activation has neuroprotective effects that alleviates SBI, suggesting that targeting this pathway could be a promising therapeutic strategy for improving patient outcome after ICH.

## Introduction

Intracerebral hemorrhage (ICH) is the most common subtype of hemorrhagic stroke, with an estimated annual incidence of 16/100,000 worldwide that is increasing with the aging population. Despite an increase in research and clinical trials for potential treatments for ICH, mortality remains high and no interventional therapy has been shown to improve patient outcome (Rodríguez-Yáñez et al., 2013; Wilkinson et al., 2017). It is generally accepted that ICH causes tissue displacement and destruction; this leads to secondary brain injury (SBI) (Qureshi et al., 2001; Schlunk and Greenberg, 2015; Behrouz, 2016), which involves a series of pathophysiological processes including activation of apoptosis (Qureshi et al., 2001; Xiong and Yang, 2015), aggravation of ischemia and edema in brain tissue surrounding the hematoma (Gebel et al., 2002), and related toxic effects (Lee et al., 2006; Chen et al., 2015). SBI can cause metabolic disorders in cells and activate stress responses including endoplasmic reticulum (ER) stress and the unfolded protein response (UPR) that either reestablish cellular homeostasis or activate cell death programs (Niu et al., 2017).

ER stress is one of the primary mechanisms that lead to apoptosis. The accumulation of misfolded/unfolded proteins induce ER dysfunction induces ER stress. Subsequently, it is induced a signal transduction cascade which is the UPR, whereby the cell tries to restore homeostasis to prevent its death (Schröder and Kaufman, 2005). The UPR is facilitated by three types of ER stress sensor proteins, protein kinase R (PKR)-like endoplasmic reticulum kinase (PERK), activating transcription factor 6 (ATF6), and inositol requiring kinase 1 (IRE1). Activated PERK phosphorylation eukaryotic translation initiation factor 2 subunit α (elF2α), which blocks most of protein translation and activates the transcription factor 4 (ATF4). PERK is a central ER stress sensor that enforces adaptive programs to recover homeostasis through a block of protein translation and the induction of the transcription factor ATF4. PERK pathway is turned off by protein phosphatase 1 (PP1) which dephosphorylates p-eIF2α (Godin et al., 2016).

The clearance of misfolded proteins by the UPR promotes neuronal survival. Thus, mild ER stress exerts neuroprotective effects by promoting autophagy (Fouillet et al., 2012), but can lead to cell death if it persists or is excessive (Tabas and Ron, 2011). The latter situation was shown to contribute to the pathophysiology of ischemia/reperfusion brain injury in rats (Nakka et al., 2010). In addition, in neurodegenerative diseases, inhibiting ER stress was found to suppress neuronal apoptosis (Moreno et al., 2013; Tsujii et al., 2015). CCAAT-enhancer-binding protein homologous protein (CHOP) is a downstream effector of the PERK pathway that functions as a pro-apoptotic factor (Jiang et al., 2012). Previous studies have shown that ER stress activates caspase-12 to induce cell apoptosis (Kim et al., 2010; Penke et al., 2016). However, and it is not known whether ER stress and apoptosis contribute to ICH-induced SBI.

The present study investigated the role of PERK signaling in SBI induced by ICH. The PERK inhibitor GSK2606414 and salubrinal, an inhibitor of eIF2α dephosphorylation, were used as experimental drugs both *in vitro* and *in vivo* (Boyce et al., 2005; Axten et al., 2012; Scheper and Hoozemans, 2013; Rubovitch et al., 2015).

## Materials and methods

### Ethical approval

All experiments were approved by the Ethics Committee of the First Affiliated Hospital of Soochow University and were performed in accordance with the guidelines of the National Institutes of Health on the care and use of animals. Adult male Sprague–Dawley (SD) rats (250–300 g) were purchased from Animal Center of Chinese Academy of Sciences, Shanghai, China. The rats were housed in temperature- and humidity-controlled animal quarters with a 12hr light/dark cycle.

### Experimental design

**Figure 1:**
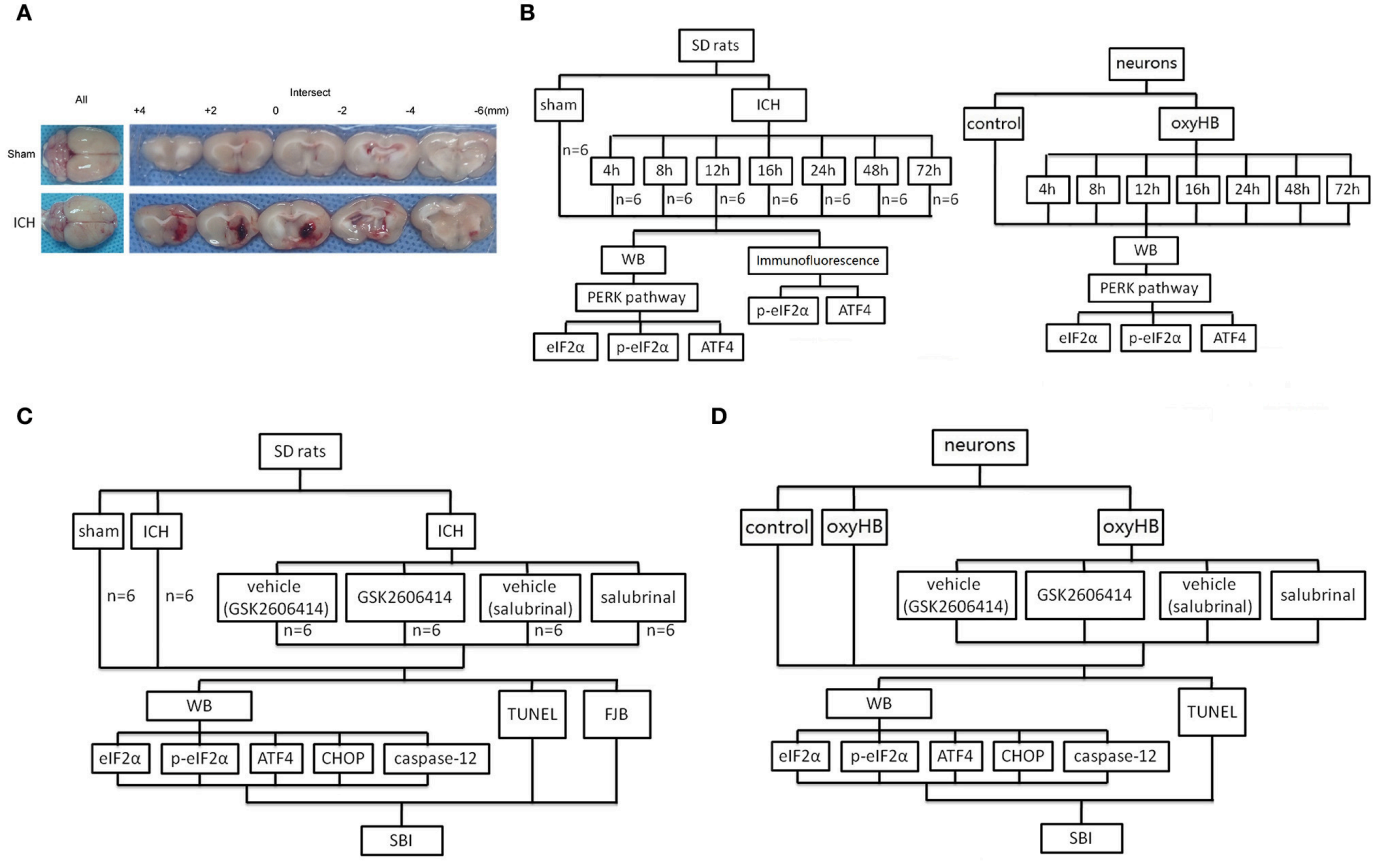
ICH model and experimental design. **(A)** Representative whole brains and brain sections from ICH model rats. **(B)** Experiment 1 evaluated the expression of eIF2α, p-eIF2α, and ATF4 *in vitro* and *in vivo*. **(C)** Experiment 2 investigated the effects of PERK signaling on ICH-induced SBI and the underlying mechanisms *in vivo*. **(D)** Experiment 3 investigated the role of PERK signaling pathway *in vitro*.

In experiment 1, 48 rats (53 rats were used, but only 48 rats survived after the surgery) were randomly assigned to eight groups of 6 rats for each, a sham group and seven experimental groups arranged by time course: 4, 8, 12, 16, 24, 48, and 72 h after ICH. The rats were euthanized at the indicated time point after ICH, and the brain tissues were separated and taken for analysis (Figure 1A). *In vitro*, the primary hippocampal neurons were assigned to eight groups, a sham group, and seven experimental groups arranged by time: 4, 8, 12, 16, 24, 48, and 72 h after neurons treated by oxyhemoglobin (OxyHb) (Figure 1B).

In experiment 2, 36 rats (43 rats were used, but only 36 rats survived) were randomly divided into six groups: sham group, ICH group, ICH +vehicle (GSK2606414) group, ICH+GSK2606414 group, ICH +vehicle (salubrinal) group and ICH +salubrinal group (*n* = 6 for each group). The administrations of drugs in each group were shown in Figure 1C. First, GSK2606414 was dissolved in dimethylsulphoxide (DMSO) to 90 μg/μl and then diluted the store solution to 90 μg/5 μl by sterile saline, which was injected intracerebroventricularly (Yan et al., 2017). Salubrinal was dissolved in DMSO to 96 μg/μl and injected intraperitoneally (1 mg/kg body weight) as reported previously (Sokka et al., 2007). Then, rats were euthanized, and the brain tissues were separated and taken for analysis (Figure 1C). The cannulated right femoral artery was used to measure blood pressure and heart rate. The blood pressure and heart rate were no significant differences among sham group, ICH group, ICH + vehicle (GSK2606414) group, ICH+GSK2606414 group, ICH +vehicle (salubrinal) group and ICH +salubrinal group (Data not shown). *In vitro*, to mimic the effect of ICH, OxyHb (10 μM) was used to treat primary hippocampal neurons (Figure 1D).

### Antibodies and drugs

Anti-CHOP antibody (ab11419), anti-p-eIF2α antibody (ab32157), anti-eIF2α antibody (ab169528), anti-XBP1 anbibody (ab37152), anti-caspase-12 antibody (ab62484), Ms mAb to NeuN (ab104224), Rb mAb to NeuN (ab177487), and anti-β-Tubulin antibody (ab179513) were purchased from abcam (Cambridge, MA, USA). Anti-ATF-4 antibody (sc-200) was purchased from Santa Cruz (Santa Cruz, CA, USA). Anti-ATF6 antibody (70B1413.1) were purchased from Novus Biological (Littleton, Co, USA). Salubrinal and GSK2606414 were purchased from TargetMol (Boston, MA, USA).

### Establishment of the ICH model

Adult male SD rats (280–330 g) were anesthetized with intraperitoneal injection of 4% chloral hydrate (0.1 mL/kg body weight). After the rats were completely anesthetized, they were fixed in the stereotactic frame (ZH-Lanxing B type, Anhui Zhenghua Biological Equipment Co. Ltd. Anhui, China). Depending on the rat's response to the pain, additional chloral hydrate should be injected. The rat is then placed on the heating pad in a supine position and the pad is maintained at a temperature of about 27–35°C. Experimental ICH model was induced by using stereotaxic insertion of autologous blood using the modified methods described by Deinsberger et al. (1996). The position of basal ganglia was 0.2 mm posterior to bregma, 3.5 mm lateral to the midline, and 5.5 mm ventral to the cortical surface. Subsequently, 100 μl of autologous blood was collected from the heart using a 100 μl microinjector (Hamilton Company, Nevada, USA). After the microinjector was in position, 100 μl of autologous blood was injected over 5 min. Typical visual representation of the brain slices from each group were shown in Figure 1A. Bone wax was used to block the drilling, and medical suture line was used to stitch the scalp. Next, put the mouse back in the cage and gave enough food and water in the cage. The assessment of SBI occurred 48 h after the onset of ICH.

### Western blot analysis

After collecting perihematomal tissues, we separately homogenized the perihematoma tissues from each experimental model. Brain homogenate was lysed in RIPA lysis buffer (Beyotime Institute of Biotechnology, Jiangsu, China). After at 16,000 g centrifuged for 5 min 4°C, the supernatant was collected. The supernatant was stored at −80°C for later use. A standard BCA (Beyotime Institute of Biotechnology) method was used to determine protein concentration. Then, a total of 50 μg protein each lane was subjected to SDS-PAGE (10%) and transferred to a membrane for ECL and imaging as reported previously (Zhai et al., 2016). The optical density was analyzed using Image J software (Rawak Software, Inc., Stuttgart, Germany).

### Immunofluorescence microscopy

The brain tissues were fixed in 4% paraformaldehyde, embedded in paraffin, cut into 4 μm sections, which was dewaxed immediately before immunofluorescence staining. Then, brain sections were stained with primary antibody, including NeuN antibody-neuronal cell marker (diluted 1:100) and antibodies for p-eIF2α (diluted 1:100), ATF-4 (diluted 1:100), at 4°C for 12 h. NEXT, brain sections were washed 3 times with PBS and stained with appropriate secondary antibodies. Normal rabbit IgG was used as negative controls for immunofluorescence assays (data not shown). Sections were observed with a fluorescence microscope (Olympus, BX50/BX-FLA/DP70, Olympus Co., Japan).

### Terminal deoxynucleotidyl transferase–mediated dUTP nick end labeling staining

Terminal Deoxynucleotidyl Transferase–Mediated dUTP Nick End Labeling (TUNEL) staining was performed as described previously to detect cell apoptosis in brain (Zhai et al., 2016). The TUNEL-positive neurons were examined and were photographed in parallel by a fluorescence microscope (Olympus, BX50/BX-FLA/DP70, Olympus Co., Japan) (3 sections per rat).

### Fluoro-jade B (FJB) staining

Fluoro-Jade B (FJB) is used to detect cell necrosis in brain tissue, which is a sensitive and highly specific fluorescent stain that reveals neuronal degradation (Zhu et al., 2014). FJB procedures were performed as previously described (Lin et al., 2012). Briefly, brain sections were deparaffinized. We used an oven to dehydrate brain sections. Then, we used xylenes and graded ethanol solutions to water for rehydrating brain sections. Brain sections were permeabilized in 0.04% Triton X-100. Next, we used FJB dye solution for incubating brain sections. Brain sections were examined and were photographed in parallel by a fluorescence microscope (Olympus, BX50/BX-FLA/DP70, Olympus Co., Japan). To evaluate the extent of cell necrosis, 6 microscopic fields in each tissue section were observed and photographed in parallel for FJB-positive cell counting. Microscopy was performed by an observer blind to the experimental condition.

### Cell culture and treatment

Primary rat cortical neurons were obtained from 17-day-old SD rat embryos as described previously (Pacifici and Peruzzi, 2012). After a week of incubation, neurons were divided into 6 groups: control, OxyHb, OxyHb + vehicle (GSK2606414), OxyHb + GSK2606414, OxyHb + vehicle (salubrinal) and OxyHb + salubrinal. To mimic ICH, in OxyHb group, neurons were treated with OxyHb (10 μM) (Zhai et al., 2016); in OxyHb + vehicle (GSK2606414) group, cells were pretreated with DMSO (volume equal to GSK2606414) for 1 h and then exposed to 10 μM OxyHb; in OxyHb + GSK2606414 group, cells were pretreated with GSK2606414 (1 μM) for 1 h and then exposed to 10 μM OxyHb (Jiang et al., 2017); in OxyHb+vehicle (salubrinal) group, cells were pretreated with DMSO (volume equal to salubrinal) for 1 h and then exposed to 10 μM OxyHb; in OxyHb +salubrinal group, cells were pretreated with salubrinal (50 μM) for 1 h and then exposed to 10 μM OxyHb (Sokka et al., 2007) in fresh medium. After incubation for 48 h, cells were fixed with 4% paraformaldehyde. After these treatments, cellular morphology was observed by inverted phase contrast microscope, and the total protein of the cells was collected and stored at −80°C for western blot analysis.

### Statistical analysis

All data are presented as means ± SEM. Graph pad prism 7 was used for all statistical analysis. One-way ANOVA for multiple comparisons and Student–Newman–Keuls *post-hoc* test were used to determine the differences among all groups. *P* < 0.05 was considered to be significant difference.

## Results

### ER stress pathways activation was induced by ICH both *in vivo* and *in vitro*

**Figure 2:**
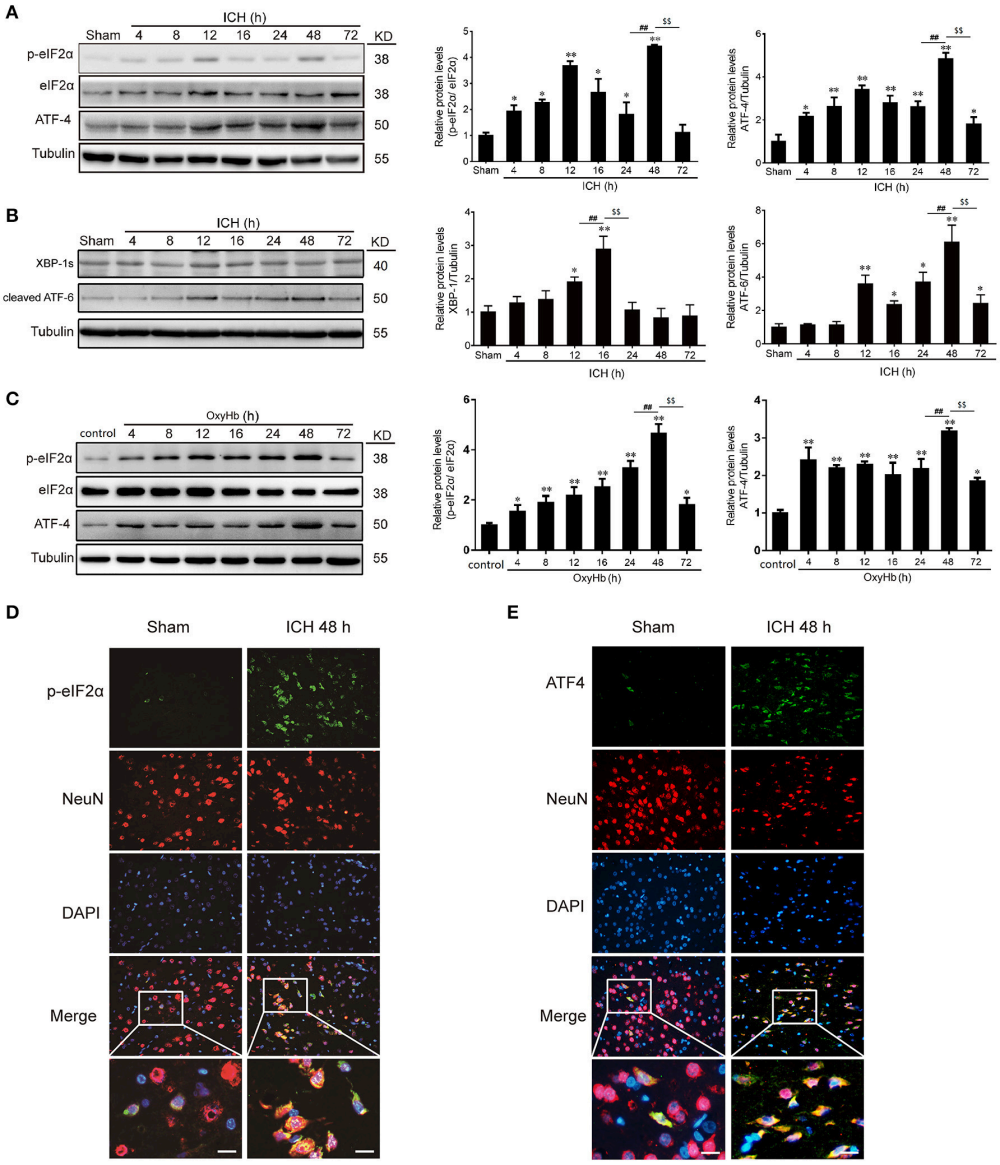
p-eIF2α and ATF4 protein levels are increased after ICH *in vitro* and *in vivo*. **(A)** Brain tissue samples collected at different time points after ICH were analyzed for p-eIF2α, eIF2α, and ATF4 expression by western blot; tubulin served as a loading control. Protein levels were quantified with ImageJ software, and mean values for sham animals were normalized to 1.0. Data represent mean ± SEM (*n* = 6). ^*^p < 0.05, ^**^p < 0.01 vs. sham; ^##^*p* < 0.01 24 vs. 48 h; ^$$^*p* < 0.01 48 vs. 72 h (one-way analysis of variance followed by the Student–Newman–Keuls *post-hoc* test). **(B)** The protein levels of ATF-6 and XBP-1 were detected by western blot, and tubulin served as a loading control. Protein levels were quantified with ImageJ software, and mean values for sham animals were normalized to 1.0. Data represent mean ± SEM (*n* = 6). ^*^p < 0.05, ^**^p < 0.01 vs. sham; ^##^*p* < 0.01 12 vs.16 h, 24 vs.48 h; ^$$^*p* < 0.01 16 vs. 24 h, 48 vs. 72 h (one-way analysis of variance followed by the Student–Newman–Keuls *post-hoc* test). **(C)** Primary neurons were extracted and treated with 10 μM OxyHb for indicated times, and p-eIF2α, eIF2α, and ATF4 levels were detected by western blotting. Protein levels were quantified with ImageJ software, and mean values in the control group were normalized to 1.0. Data represent mean ± SEM (*n* = 3). ^*^p < 0.05, ^**^p < 0.01 vs. control; ^##^*p* < 0.01 24 vs. 48 h; ^$$^*p* < 0.01 48 vs. 72 h (one-way analysis of variance followed by the Student–Newman–Keuls *post-hoc* test). **(D,E)** Double immunofluorescence analysis of brain tissue (between the cortex and the perihematoma) using antibodies against eIF2α (green) and NeuN (red) **(D)** or ATF4 (green) and NeuN (red) **(E)**; nuclei were labeled with DAPI (blue). Scale bar = 30 μm.

In experiment 1, the western blot analysis revealed that the levels of p-eIF2α and ATF-4 in the brain tissues were increased significantly from 4 h after ICH, reaching a peak at 48 h (Figure 2A). In addition, we also determined the other two ER stress pathways, and it was shown that ATF-6 and XBP-1 were elavated after ICH induction, exhibiting that ATF and IREα/XBP1 were also activated (Figure 2B). *In vitro*, to mimic ICH we used OxyHb to treat the neurons. And it was found that the level of p-eIF2α and ATF-4 were increased significantly from 4 h after OxyHb treatment and reached the peak at 48 h, as expected (Figure 2C). Furthermore, double immunofluorescence assay verified the ICH-induced increase in the protein level of p-eIF2α and ATF-4 in neurons at 48 h, which were also demonstrated that p-eIF2α and ATF-4 were mainly expressed in neurons (Figures 2D,E). So, we focused on PERK pathway in neurons in the following study at 48 h.

### PERK signaling pathway was inhibited by GSK2606414 and activated by salubrinal *in vivo*

**Figure 3:**
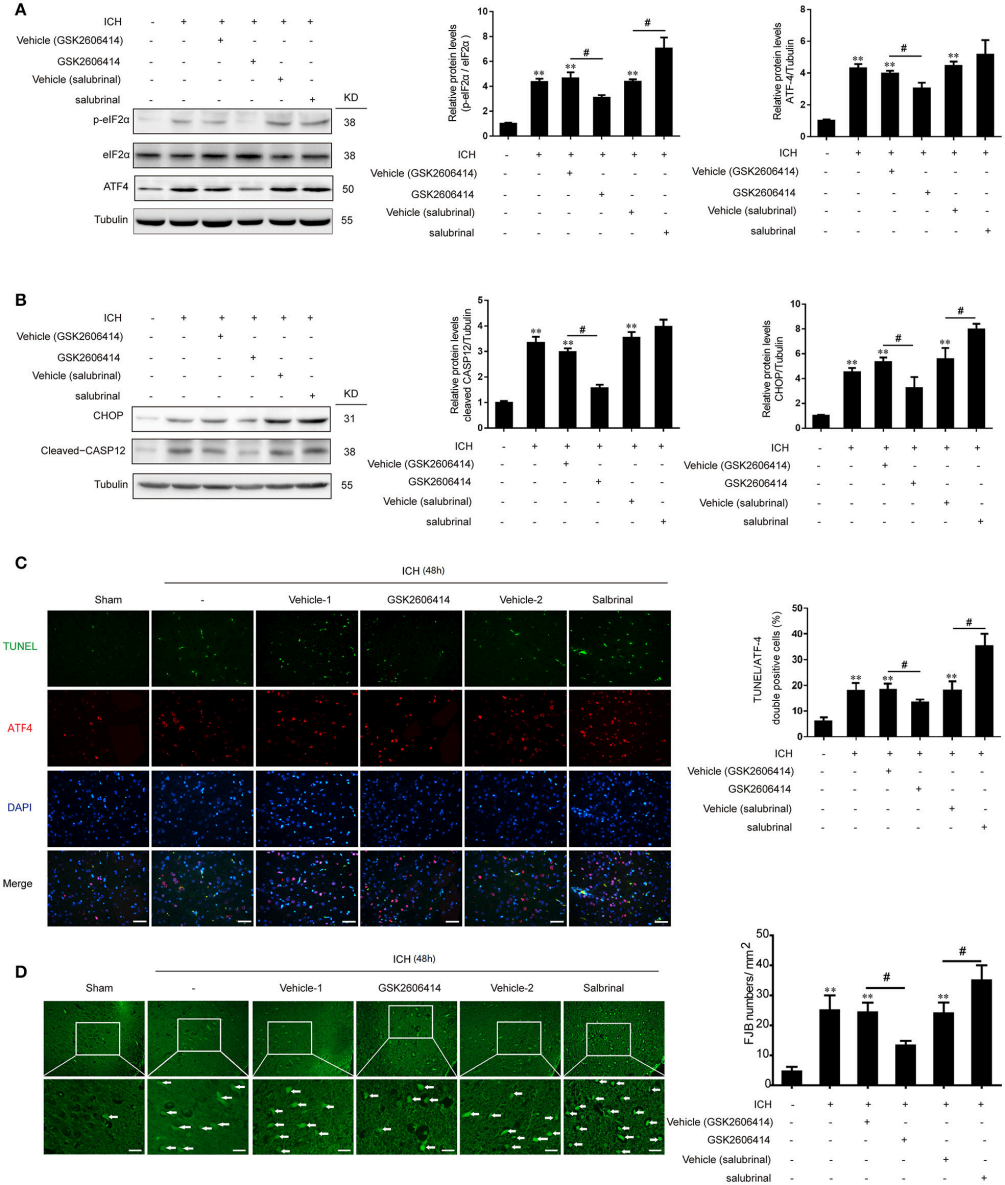
Effect of PERK pathway inhibition and activation on SBI following ICH *in vivo*. **(A)** Brain tissue samples were collected and eIF2α phosphorylation and ATF4 expression were detected by western blotting. Tubulin served as a loading control. Protein levels were quantified with ImageJ software. Mean values for the sham group were normalized to 1.0. Data represent mean ± SEM (*n* = 6). ^**^p < 0.01 vs. sham; ^#^*p* < 0.05 vs. indicated vehicle (one-way analysis of variance followed by the Student–Newman–Keuls *post-hoc* test). **(B)** Detection of CHOP and caspase-12 expression in sham, ICH, ICH + vehicle (GSK2606414), ICH + GSK2606414, ICH + vehicle (salubrinal), and ICH + salubrinal groups 48 h after ICH by western blotting. Data represent mean ± SEM (*n* = 6). ^**^p < 0.01 vs. sham; ^#^*p* < 0.05 vs. indicated vehicle. **(C)** Induction of apoptosis 48 h after ICH, as detected with the TUNEL assay. Double immunofluorescence analysis was performed with TUNEL (green) and an antibody against ATF-4 (red); nuclei were labeled with DAPI (blue). Scale bar = 30 μm. Quantitative analysis of TUNEL and ATF-4 double positive neurons in each group. Data represent mean ± SEM (*n* = 6). ^**^p < 0.01 vs. sham; ^#^*p* < 0.05 vs. indicated vehicle. **(D)** Detection of neuronal degradation in the cerebral cortex by FJB staining (green). Scale bar = 26 μm. Arrows indicate FJB-positive cells. FJB-positive cells/mm^2^ was quantified at 48 h. Data represent mean ± SEM (*n* = 6). ^**^p < 0.01 vs. sham; ^#^*p* < 0.05 vs. indicated vehicle.

The PERK inhibitor GSK2606414 was injected intracerebroventricularly at 1 h after ICH and the eIF2α dephosphorylation inhibitor salubrinal, as an agonist of PERK downstream signaling pathway, was injected intraperitoneally at 30 min before ICH, respectively. It was revealed that with the treatment of GSK2606414 and salubrinal, the protein levels of p-eIF2α and ATF-4 were decreased and increased compared with ICH + vehicle (GSK2606414) and ICH + vehicle (salubrinal) group respectively (Figure 3A).

### PERK pathway promoted ICH-induced apoptosis *in vivo*

Previous studies have shown that PERK signaling pathway was involved in ER stress-induced apoptosis. In the present study, we found that with the treatment of GSK2606414, the increase of CHOP and cleaved caspase-12 protein levels induced by ICH could be significantly reversed (Figure 3B). Meanwhile, as indicated in the histological evidence of neuronal apoptosis, the TUNEL was double labeled with ATF-4. The results showed that the number of TUNEL and ATF-4 double positive cells was increased following ICH relative to the sham group, but this effect was abrogated by GSK2606414 administration (Figure 3C). Similarly, the ICH-induced increase in the number of FJB-positive cells was reversed by GSK2606414 as compared to the ICH + vehicle (GSK2606414) group (Figure 3D). On the contrary, the salubrinal treatment could significantly promote the protein levels of CHOP and cleaved caspase-12 increase induced by ICH (Figure 3B). Moreover, the TUNEL and ATF-4 double positive cells were significantly increased compared to the ICH+vehicle (salubrinal) group (Figure 3C), as well as the FJB-positive cells (Figure 3D). It was indicated that PERK pathway inhibition could rescue neuronal apoptosis and necrosis induced by ICH.

### PERK signaling pathway was inhibited by GSK2606414 and activated by salubrinal *in vitro*

**Figure 4:**
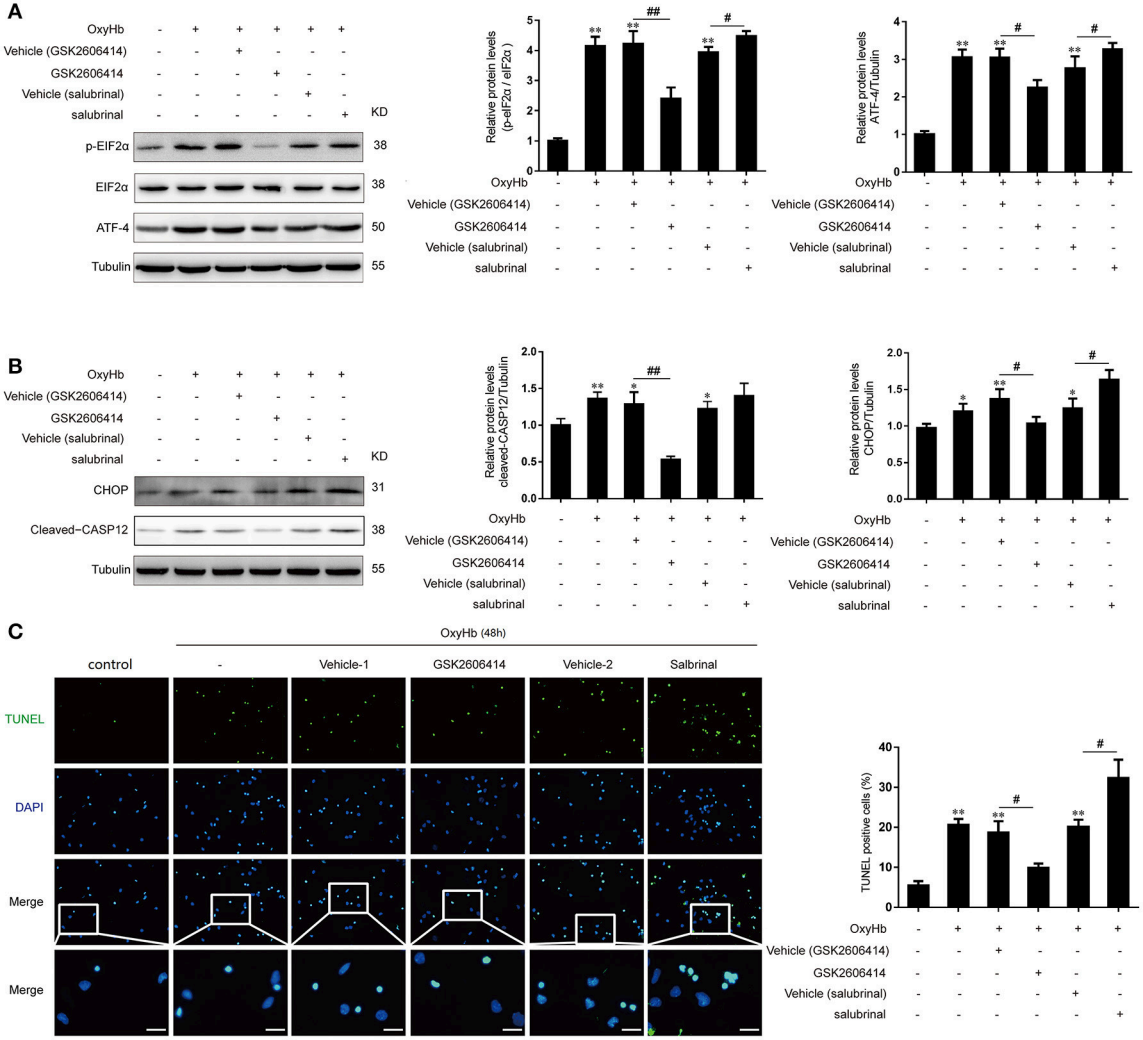
Effect of PERK pathway inhibition and activation on OxyHb-induced neuronal apoptosis *in vitro*. Neurons were cultured with or without OxyHb for 48 h. Cells were exposed to GSK2606414 or salubrinal for 1 h before OxyHb treatment. **(A)** eIF2α phosphorylation and ATF4 expression in the control, OxyHb, OxyHb + vehicle (GSK2606414), OxyHb + GSK2606414, OxyHb + vehicle (salubrinal), and OxyHb + salubrinal groups were detected by western blotting. Data represent mean ± SEM (*n* = 3). ^**^p < 0.01 vs. control; ^#^*p* < 0.05, ^##^*p* < 0.01 vs. indicated vehicle. **(B)** CHOP and cleaved-caspase-12 expression in each group was detected by western blotting. Data represent mean ± SEM (*n* = 3). ^*^p < 0.05, ^**^p < 0.01 vs. control; ^#^*p* < 0.05, ^##^*p* < 0.01 vs. indicated vehicle. **(C)** Apoptosis in OxyHb-treated neurons at 48 h was detected with the TUNEL assay. Representative images from control, OxyHb, OxyHb + vehicle (GSK2606414), OxyHb + GSK2606414, OxyHb + vehicle (salubrinal), and OxyHb + salubrinal groups are shown. Scale bar = 20 μm. The percentage of TUNEL-positive cells was determined. Data represent mean ± SEM (*n* = 3). ^**^p < 0.01 vs. control; ^#^*p* < 0.05 vs. indicated vehicle.

In addition, we further investigated the role of PERK signaling pathway in primary neurons treated with OxyHb to mimic ICH. It was found that the protein levels of p-eIF2α and ATF-4 were significantly decreased after GSK2606414 treatment compared to the OxyHb + vehicle (GSK2606414) group, which showed the opposite effects with the treatment of salubrinal compared to the OxyHb +vehicle (salubrinal) group (Figure 4A).

### PERK signaling pathway promoted ICH-induced neuronal apoptosis *in vitro*

Similar to the results obtained in the *in vivo* experiments, with the treatment of OxyHb, the CHOP and cleaved-caspase-12 protein levels were significantly elevated, indicating that apoptosis induction (Figure 4B). Importantly, after the treatment of GSK2606414 and salubrinal, the protein levels of CHOP and cleaved-caspase-12 were significantly decreased and increased, respectively compared to the OxyHb + vehicle (GSK2606414) group and the OxyHb + vehicle (salubrinal) group respectively (Figure 4B). Accordingly, the number of TUNEL-positive primary neurons was decreased by GSK2606414 following OxyHb pretreatment relative to the OxyHb + vehicle (GSK2606414) group, while the opposite was observed in the OxyHb + salubrinal group as compared to the vehicle control (Figure 4C). These results demonstrate that the PERK pathway plays an important role in ICH-induced neuronal apoptosis.

## Discussion

**Figure 5:**
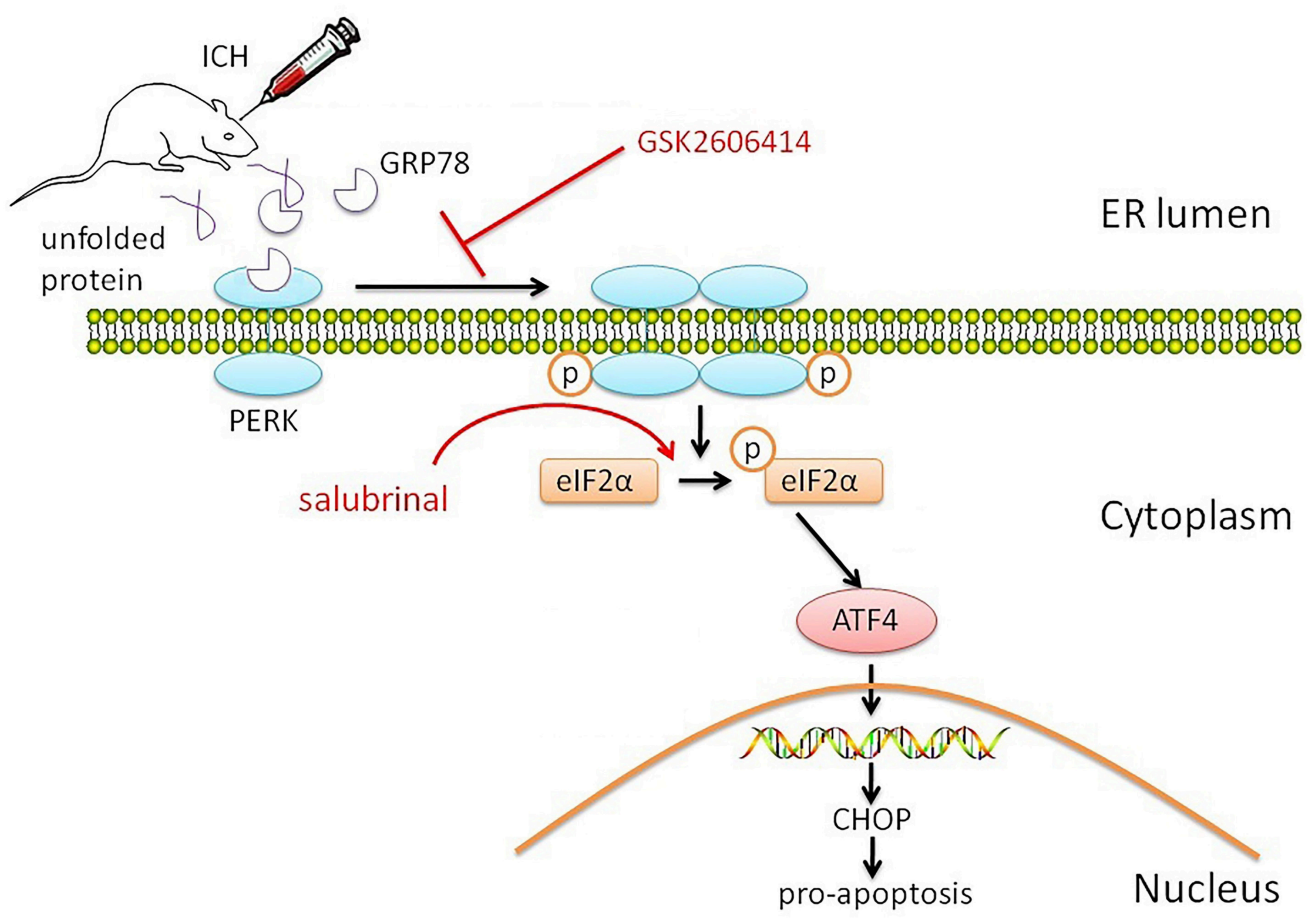
Proposed role of PERK signaling pathway in SBI after ICH. The PERK signaling pathway is activated by ICH, resulting in increased p-eIF2α and ATF4 protein levels. The consequent activation of the ER stress response induces neuronal apoptosis, which is blocked by application of the PERK inhibitor GSK2606414. On the contrary, activation of PERK downstream signaling pathway by salubrinal promotes apoptosis and reduces neuronal survival by blocking eIF2α dephosphorylation.

ICH is followed by brain injury that can disrupt cell metabolism and activate cellular stress responses, including the UPR and ER stress (Niu et al., 2017). In this study, we investigated the role of PERK signaling in the pathophysiology of SBI following ICH and found that the PERK pathway was activated, as evidenced by increased protein levels of p-eIF2α and ATF4. The resultant ER stress induced neuronal apoptosis (Figure 5).

Disruption of ER function leads to ER stress (Roussel et al., 2013). Oxidative stress (Goswami et al., 2016), mitochondrial calcium overload (Zhou et al., 2015), perturbation of cellular ion balance (Varadarajan et al., 2013), and toxic glutamate release (Li et al., 2015) have been shown to induce ER stress in various diseases. The PERK pathway plays an important role in neuronal fate as an important mediator of ER stress. ER stress-associated PERK/eIF2α signaling is activated in response to elevated levels of misfolded proteins in the ER and temporarily halts protein translation, which can lead to neuronal death (Li et al., 2015; Radford et al., 2015). PERK/eIF2α signaling is increased in cerebral ischemia (Gharibani et al., 2015), and the constituent proteins have been shown to be upregulated in neurons upon central nervous system injury (Han et al., 2015; Rubovitch et al., 2015; Yan et al., 2017). Consistent with these observations, we found here that p-elF2α and ATF4 levels were significantly elevated in neurons after ICH, with maximum levels observed after 48 h both *in vitro* and *in vivo*.

As a mediator of ER stress, PERK signaling is involved in neuronal apoptosis after subarachnoid hemorrhage; PERK is inhibited by Akt-associated anti-apoptotic pathways, which reduces early brain injury (Yan et al., 2017). PERK and eIF2α levels are elevated in traumatic brain injury (Rubovitch et al., 2015). Severe ER stress leads to apoptosis, while inhibition of ER stress promotes neuronal survival and improves neurological function (Moreno et al., 2013; Rubovitch et al., 2015; Tsujii et al., 2015). In accordance with previous studies, we found that GSK2606414 suppressed p-eIF2α and ATF4 expression and promoted neuronal survival by suppressing apoptosis 48 h after ICH. Additionally, PERK inhibition decreased CHOP and cleaved caspase-12 levels. Thus, inhibiting PERK signaling has a neuroprotective effect following ICH. Indeed, GSK2606414 was shown to exert neuroprotective effects in tauopathies and Parkinson's and Alzheimer's diseases as a selective inhibitor of PERK (Halliday et al., 2015; Radford et al., 2015).

Some studies have suggested that increased PERK activation can reduce neuronal apoptosis in various diseases (Fouillet et al., 2012; Lin et al., 2013). There is no consensus on whether ER stress and UPR are beneficial or detrimental following central nervous system injury, and the role of PERK signaling in SBI after ICH remains unclear. Previous studies have shown that a mild stimulus can activate ER stress as a host defense mechanism, resulting in the degradation of damaged organelles and proteins by autophagy, which promotes neuronal survival (Fouillet et al., 2012; Yan et al., 2014). ER stress cannot counter stimuli that are severe and long-lived, resulting in apoptosis (Moreno et al., 2013; Tsujii et al., 2015). Perihematomal edema and the physiological response to hematoma after ICH can cause SBI (Aronowski and Zhao, 2011; Urday et al., 2015). Blood components, dysfunctional organelles, overproduced iron complexes, and cytokine levels continuously increase following ICH, resulting in disruption of normal protein folding and activation of ER stress and the UPR, which contribute to ICH-associated brain injury (Guo et al., 2012). In humans, intracerebral hematoma resolves gradually over a period of weeks, during which time the brain experiences continuous injury (Keep et al., 2012), leading to prolonged and severe ER stress and eventually neuronal apoptosis.

Also, there are a few limitations to this study. Firstly, in this study, we only focused exclusively on the role of PERK signaling pathway in adult male rats although ICH can affect females and is common in the elderly (Tsivgoulis et al., 2014). Secondly, a previous study has shown that PERK pathway via direct interaction to promote the enzymatic activity of calcineurin (Gao et al., 2016). Calcium overload in the cytoplasm is thought to be a potential mechanism of apoptosis induced by calcineurin; therefore, the precise relationship between PERK and calcineurin merits closer examination in future studies.

In conclusion, the results of this study demonstrate that PERK signaling pathway inhibition can reduce SBI after ICH by suppressing apoptosis. Based on these findings, we propose that PERK signaling pathway could be a key endogenous physiological regulatory signal pathway in neurons, suggesting that it might be a therapeutic target to alleviate SBI following ICH.

## Author contributions

ZW and XL: Conceived and designed the study, including quality assurance and control; CM and JZ: Performed the experiments and wrote the paper; BD and HS: Designed the study's analytic strategy; XL and HL: Helped conduct the literature review and prepare the Materials and Methods section of the text. All authors read and approved the manuscript.

### Conflict of interest statement

The authors declare that the research was conducted in the absence of any commercial or financial relationships that could be construed as a potential conflict of interest.
